# Pro-Resolving Effect of Ginsenosides as an Anti-Inflammatory Mechanism of *Panax ginseng*

**DOI:** 10.3390/biom10030444

**Published:** 2020-03-13

**Authors:** Dong-Soon Im

**Affiliations:** 1 Laboratory of Pharmacology, College of Pharmacy, Kyung Hee University, 26 Kyungheedae-ro, Dongdaemun-gu, Seoul 02447, Korea; imds@khu.ac.kr; Tel.: +82-2-961-9377; Fax: +82-2-961-9580; 2 Department of Life and Nanopharmaceutical Sciences, Graduate School, Kyung Hee University, 26 Kyungheedae-ro, Dongdaemun-gu, Seoul 02447, Korea

**Keywords:** ginsenoside, anti-inflammation, pro-resolving, ginseng, macrophage, M2 polarization

## Abstract

*Panax ginseng*, also known as Korean ginseng, is a famous medicinal plant used for the treatment of many inflammatory diseases. Ginsenosides (ginseng saponins) are the main class of active constituents of ginseng. The anti-inflammatory effects of ginseng extracts were proven with purified ginsenosides, such as ginsenosides Rb1, Rg1, Rg3, and Rh2, as well as compound K. The negative regulation of pro-inflammatory cytokine expressions (TNF-α, IL-1β, and IL-6) and enzyme expressions (iNOS and COX-2) was found as the anti-inflammatory mechanism of ginsenosides in M1-polarized macrophages and microglia. Recently, another action mechanism emerged explaining the anti-inflammatory effect of ginseng. This is a pro-resolution of inflammation derived by M2-polarized macrophages. Direct and indirect evidence supports how several ginsenosides (ginsenoside Rg3, Rb1, and Rg1) induce the M2 polarization of macrophages and microglia, and how these M2-polarized cells contribute to the suppression of inflammation progression and promotion of inflammation resolution. In this review, the new action mechanism of ginseng anti-inflammation is summarized.

## 1. Introduction—Ginseng

**Figure 1:**
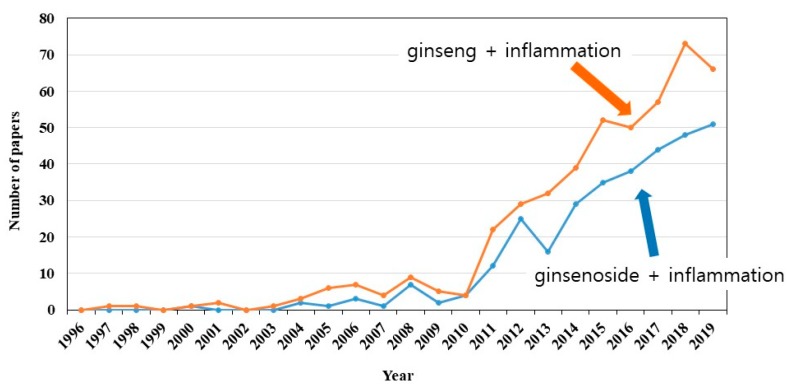
Annual changes in the number of published papers on ginseng, ginsenoside, and inflammation.

Ginseng, the root of *Panax ginseng* C.A. Meyer, was used for thousands of years as a tonic herb that provides numerous benefits in Asian countries like Korea and China [1]. As *Panax* means “heals all” in Greek, the effects of ginseng are the longevity and replenishment of vital energy in traditional Chinese medicine [1,2,3]. In the modern era, ginseng attracts great interest because of its various pharmacological and therapeutic effects on aging, cancer, the cardiovascular system, diabetes, immune-regulatory function, and inflammation [4,5,6,7,8]. There are various components in ginseng, including ginsenosides, gintonin, polysaccharides, polypeptides, glycoconjugate compounds, and other compounds [1]. Over the last decade, extensive studies elucidated ginsenosides (ginseng saponins) as the chief active constituents of ginseng, especially with regard to anti-inflammatory effects [1]. As shown in Figure 1, many papers with two keywords in their abstracts were retrieved, i.e., ginseng plus inflammation (658 papers) or ginsenoside plus inflammation (274 papers), in a public database, PubMed (https://www.ncbi.nlm.nih.gov/pubmed). Since 2010, the number of papers on ginsenosides and inflammation increased.

## 2. Ginsenosides in Anti-Inflammation

Inflammation is part of the immunological response in the body to infection or injury, and it is associated with numerous human diseases and conditions [9]. A dynamic balance between pro-inflammatory cytokines (TNF-α and IL-1β) and anti-inflammatory cytokines (IL-2, IL-4, and IL-10) modulates the status of inflammation, while an imbalance or overwhelming production of pro-inflammatory cytokines subsequently results in inflammation-related diseases such as diabetes, cancer, cardiovascular disease, and neurological diseases [10,11]. Inflammation is also essential in the process of repairing tissue and restoring tissue homeostasis [10,11].

Ginsenosides—dammarane-type triterpene glycosides—are the representative active ingredients of ginseng [2]. Almost 100 different types of ginsenosides were isolated from the roots of Korean and American ginseng [12]. Ginsenosides are expressed by Rx, where x is determined by the distance from the origin of thin-layer chromatography [10]. The most polar segment is marked as A and the least polar one is marked as H [10]. Ginsenosides are generally divided into three groups: protopanaxadiols, protopanaxatriols, and oleanane (ginsenoside Ro). Protopanaxadiols have sugar moieties on the C-3 position of dammarane-type triterpene, such as ginsenosides Rb1, Rb2, Rb3, Rc, Rd, Rg3, Rh2, and Rh3. Protopanaxatriols have sugar moieties on the C-6 position of dammarane-type triterpene, such as ginsenosides Re, Rf, Rg1, Rg2, and Rh1 [10,11,13].

**Figure 2:**
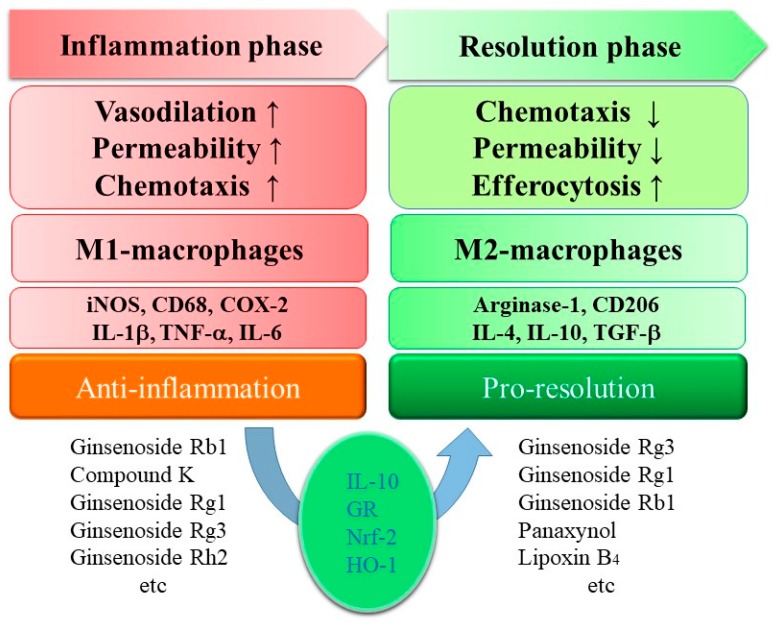
Summarized effects of ginsenosides on M1- and M2-polarized macrophages during inflammatory and resolving phases.

The anti-inflammatory effects of ginseng extracts were proven with purified ginsenosides. The negative regulation of pro-inflammatory cytokine expressions (TNF-α, IL-1β, and IL-6) and enzyme expressions (iNOS and COX-2) was found as the anti-inflammatory mechanism of ginsenosides in M1-polarized macrophages and microglia (Figure 2) [10,11]. Among them, the most commonly studied ginsenosides are Rb1, Rg1, Rg3, Re, Rd, and Rh1 [11]. Kim et al. published a review on the role of ginsenosides in inflammatory responses and diseases [10]. The reported pharmacology and signal transduction are well summarized for each ginsenoside, i.e., ginsenosides Rb1, Rb2, Rd, Re, Rg1, Rg3, Rg5, Rh1, Rh2, and Rp1, sulfated Rh2, and compound K [10]. This paper provides an update on the more anti-inflammatory ginsenosides, such as ginsenosides Rc, Rf, Rg5, Rg6, Rh3, Rk1, Ro, and Rz1, as well as ginseng glycopeptides, and it also summarizes a mechanistic viewpoint in anti-inflammatory ginseng pharmacology.

Ginsenoside Rc was found to show the highest inhibitory activity against the expression of TNF-α, IL-1, and IFNs, and it attenuated inflammatory symptoms in type II collagen-induced arthritis, ethanol/HCl-mediated gastritis, and LPS/d-galactosamine-triggered hepatitis [14]. Ginsenoside Rc was also found to exert anti-inflammatory actions by means of suppressing TANK-binding kinase 1/IκB kinase ε/interferon regulatory factor-3 and p38/ATF-2 signaling [15]. Furthermore, ginsenoside Rc significantly enhanced glucose uptake in C2C12 myotubes by inducing ROS generation, which leads to AMPK and p38 MAPK activation, suggesting its potential as an anti-diabetic agent [16]. Later, Kim et al. found that ginsenoside Rc modulates forkhead box O (FoxO1) phosphorylation through the activation of PI3K/Akt and inhibition of AMPK and FoxO1 acetylation, leading to an upregulation of catalase under conditions of oxidative stress in HEK293 cells [17].

Ginsenoside Rf showed an inhibitory effect on the inflammatory mediators downstream of p38/NF-κB activation, such as the reduction of IL-1β, IL-6, TNF-α, NO, and ROS productions, on TNF-α-stimulated HT-29 intestinal epithelial cells and RAW264.7 mouse macrophage cells [18]. Moreover, its anti-inflammatory activity, along with ginsenoside Rb1 and Rg1, was reported, and anti-oxidation and the inhibition of NO synthesis were proposed as its mechanism [19,20].

Ginsenoside Rf could significantly attenuate Aβ-induced apoptosis in N2A cells, accelerate Aβ clearance, and reduce Aβ level in N2A cells stably transfected with human Swedish mutant APP695 [21]. Daily treatment with ginsenoside Rf improved spatial learning and memory in an Aβ_42_-induced mouse model of Alzheimer’s disease [21]. In a surgically induced rat endometriosis model, ginsenoside Rf could decrease the volume of endometriotic implants and the writhing response [22]. Expression levels of VEGF and inflammation-related iNOS, IL-6, IL-1β, and TNF-α were significantly downregulated in the ginsenoside Rf-treated group in a dose-dependent manner [22]. In a rat nerve injury-induced neuropathic pain model, chronic ginsenoside Rf treatment partially reversed the upregulation of pro-inflammatory cytokines in the spinal cord and/or the dorsal root ganglion, but elevated IL-10, an anti-inflammatory factor [23].

Ginsenoside Rg6, a rare ginsenoside from ginseng, was found to have a significant immunosuppressive function on TLR4-induced systemic inflammatory responses, i.e., LPS-induced septic shock, cecal ligation and puncture-induced sepsis [24]. Mechanistically, ginsenoside Rg6 augmented IL-10 expression in bone marrow-derived macrophages, whereas it inhibited NF-κB activation and MAP kinases via induction of miR-146a, an operator microRNA (miRNA) for anti-inflammation [24].

Ginsenosides Rz1, Rk1, and Rg5 were present in heat-treated ginseng in a ratio of 1:2:6 [25]. These converted ginsenosides from primary protopanaxdiol ginsenosides significantly inhibited COX-2 and iNOS gene expression and inhibited TNF-α-induced NF-κB expression [26].

Ginsenoside Rk1 was studied as a mixture form with ginsenoside Rg5 in a 1:1 weight ratio for its effects on atopic dermatitis. In the study, the mixture of ginsenoside Rg5:Rk1 attenuated TNF-α/IFN-γ-induced phosphorylation of p38 MAPK, STAT1, and NF-κB/IKKβ in HaCaT cells and decreased LPS-mediated NO and ROS production in RAW264.7 macrophages [27]. Ginsenoside Rk1 also inhibited LPS-induced expression of NO, IL-6, IL-1β, TNF-α, and MCP-1 by means of blocking the activation of NF-κB and the Jak2/Stat3 pathway in RAW264.7 cells [28]. Ginsenoside Rk1 was also found to exhibit a strong inhibitory effect on arachidonic acid-induced platelet aggregation [29]. Decreased productions of thromboxane B_2_, a key element in platelet aggregation, and 12-hydroxy-5,8,10,14-eicosatetraenoic acid (12-HETE), an arachidonic acid metabolite, were observed in the ginsenoside Rk1-treated platelets via inhibition of COX activity and 12-lipoxygenase translocation resulting from decreased Ca^2+^ levels [29].

Ginsenoside Ro, an oleanane-type saponin, inhibited an increase in vascular permeability in mice, induced by acetic acid, and it reduced acute paw edema in rats induced by compound 48/80 or carrageenan, without suppressing edema in arthritic rats [30]. In experimental models of acute and chronic hepatitis, ginsenoside Ro inhibited the increase of serum AST and ALT levels in d-galactosamine- and CCl_4_-induced acute hepatitic rats [31]. Ginsenoside Ro could also suppress IL-1β-induced apoptosis of rat chondrocytes by inhibiting levels of Bax and Bad, decreasing p53 phosphorylation, and promoting the expression of Bcl-xL and PCNA [32]. Ginsenoside Ro also alleviated IL-1β-induced inflammation and matrix degradation by downregulating the expression of MMP 3, MMP 9, and COX-2, and inhibited NF-κB p65 phosphorylation, suggesting its potential for the treatment of osteoarthritis [32]. Recently, ginsenoside Ro was found to decrease inflammatory NO synthase and COX-2 expression induced by LPS and to increase the expression of heme oxygenase-1 (HO-1) in a dose-dependent manner in RAW264.7 macrophages [33].

Ginseng glycopeptides were tested in inflammatory pain models induced by carrageenan and rat pain models induced by Faure Marin [34]. Glycoproteins extracted from ginseng have a molecular weight in the range of 0.4 to 4.4 kDa. Significant differences were found in IL-1β, IL-2, IL-4, TNF-α, and histamine via the treatment of glycoproteins. In the Morris water maze test, the glycopeptides effectively alleviated the memory impairment symptoms of rats induced by Aβ_25–35_, and they showed significant protective activity against the apoptosis of SH-SY5Y neuronal cells induced by Aβ_25–35_ [35].

For the mechanism of ginseng anti-inflammation, several targets were proposed: (1) activation of the glucocorticoid receptor, the target of steroidal anti-inflammatory drugs such as cortisol and dexamethasone (compound K, ginsenosides Rg1 and Re) [36,37,38,39]; (2) an anti-oxidation-related mechanism, i.e., inhibition of ROS production and activation of Nrf-2 and HO-1 (compound K, ginsenosides Rg1, Rb1, Ro, and Rg5) [27,33,36,40,41,42]; (3) blocking of TLR4 interaction with LPS (ginsenosides Re and Rg5) [43,44]; (4) activation of anti-inflammatory PPARγ (ginsenosides Rg3, Re, Rb1, Rg1, and Rf) [40,45,46,47,48,49,50].

## 3. Ginsenosides in Pro-Resolution

Macrophages that are widely distributed play an indispensable role in homeostasis and defense as part of the immune system [51,52]. They can be polarized phenotypically by the microenvironment to mount specific functional programs [51,52]. The polarization of mononuclear phagocytes is a useful simplified conceptual framework, describing a continuum of functional states classified according to their phenotypes [51,52]. Classically activated macrophages are called M1-polarized macrophages. Prototypical stimuli are IFN-γ and LPS. Alternatively activated macrophages are called M2-polarized macrophages. Depending on the stimuli, they are subdivided into M2a, M2b, and M2c, induced after exposure to IL-4 or IL-13 (M2a), to immune complexes in combination with IL-1β or LPS (M2b), and to IL-10, TGF-β, or glucocorticoids (M2c), respectively [51,52]. M2-polarized macrophages play a role in the resolution of inflammation through high endocytic clearance capacities and trophic factor synthesis, as well as reduced pro-inflammatory cytokine production (Figure 2) [51,52]. Resolution of inflammation is now considered to be an active process driven by M2-polarized macrophages [53,54]. There are three constituents in ginseng reported to drive M2 polarization, i.e., ginsenosides Rg3, Rb1, and Rg1.

Based on the induction of M2 macrophage polarization, ginsenoside Rg3 was identified as a pro-resolving ginsenoside. Ginsenoside Rg3 not only induced the expression of arginase-1 (a representative M2 marker gene), but also suppressed M1 marker genes, such as inducible NO synthase and NO levels [55]. Previously, anti-inflammatory effects of ginsenoside Rg3 were reported in M1 activated macrophages. Ginsenoside Rg3 suppressed NO, ROS, and prostaglandin E_2_ (PGE_2_) productions induced by LPS in RAW264.7 macrophages in a concentration-dependent manner [56]. Moreover, ginsenoside Rg3 suppressed matrix MMP 9 activity, COX-2 expression, and pro-inflammatory cytokine production, such as TNF-α, IL-1β, and IL-6 [56]. Similarly, enhanced ginsenoside Rg3 significantly suppressed the expression of IFN-γ and TBX21 in T cells under Th1-skewing conditions [57]. Furthermore, oral administration of enhanced ginsenoside Rg3 suppressed the frequency of Th1 cells in the Peyer’s patch and lamina propria cells in vivo [57]. Ginsenoside Rg3-enriched red ginseng extract potently suppressed NO production in murine RAW 264.7 macrophages, without any cytotoxicity across dosages. Additionally, it inhibited the mRNA expression of pro-inflammatory mediators and cytokines such as iNOS, COX-2, IL-1β, IL-6, and TNF-α [58]. Therefore, ginsenoside Rg3 was reported as an anti-inflammatory constituent in M1-polarized macrophages and in vivo conditions. Recently, ginsenoside Rg3 induction of M2 polarization in mouse peritoneal macrophages was initially reported by Kang et al. among 11 tested ginsenosides (Rb1, Rb2, Rc, Rd, Re, Rf, Rg1, Rg2, Rg3, Rh2, and Ro) [55].

In a zymosan-induced peritonitis model, the pro-resolving activity of ginsenoside Rg3 was confirmed in vivo [55]. When ginsenoside Rg3 was administrated at peak inflammatory response (12 h after zymosan treatment) into the peritoneal cavity, it accelerated the resolution process, i.e., the rapid disappearance of immune cells. Therefore, ginsenoside Rg3 induces the M2 polarization of macrophages in vitro and accelerates the resolution of inflammation in vivo [55]. Ginsenosides Rg1 and Rh2 were also reported to induce arginase-1 expression in the peritoneal macrophages in a concentration-dependent manner up to 5 μM [55].

In another study, Guo et al. showed a similar observation [59]. Treatment of advanced glycation end products promoted the expression of M1 markers (iNOS and CD86) and pro-inflammatory molecules, whereas ginsenoside Rg3 reversed the M1 polarization to the M2 phenotype expressing arginase 1 and CD206 (i.e., mannose receptor), two M2 markers in vitro [59]. The administration of ginsenoside Rg3 promoted atherosclerotic plaque stability, which was accompanied by increased M2 phenotype macrophages and reduced M1 phenotype macrophages in the plaque [59]. By means of a PPARγ antagonist, GW9662, the important role of PPARγ pathways was suggested in mediating ginsenoside Rg3 effects in macrophage polarization and atherosclerotic plaque stability [59].

Well-known pro-resolving lipids are arachidonic acid-derived lipoxins (lipoxin B_4_ and lipoxin A_4_) and ω-3 polyunsaturated fatty acid-derived resolvins, protectin, and maresins [53,54]. Previously, ginsenoside Rg3 was reported to increase the level of lipoxin B_4_ and to decrease various prostaglandins and HETEs, implying the pro-resolution and anti-inflammatory action of ginsenoside Rg3 [56].

Treatment with ginsenoside Rb1 induced expression of the classic M2 macrophage markers (arginase-1 and CD206), while expression of the M1 macrophage marker, iNOS, was suppressed in primary peritoneal macrophages [60]. Ginsenoside Rb1-induced M2 polarization was found to be achieved partly by the production of IL-4 and/or IL-13 and STAT6 phosphorylation [60]. In an ApoE-deficient atherosclerosis model, the administration of ginsenoside Rb1 increased the M2 macrophage phenotype in atherosclerotic plaque and promoted atherosclerotic lesion stability [60].

Ginsenoside Rg1 significantly improved chemotherapy-induced cognitive impairment-like behavior in the water maze test and suppressed chemotherapy-induced elevation of the pro-inflammatory cytokines TNF-α and IL-6 [61]. In addition, it increased the levels of the anti-inflammatory cytokines IL-4 and IL-10 in multiple sera and brain tissues, and it also inhibited chemotherapy-induced microglial polarization from M2 to M1 phenotypes [61]. Chemotherapy caused an increase in IL-6-labeled M1 microglia, but a decrease in the expression of arginase 1-labeled M2 microglia in both brain tissues and cultured microglial cells [61]. However, ginsenoside Rg1 co-treatment inhibited microglial polarization from M2 to M1 phenotype [61], supporting the observation of peritoneal macrophage M2 polarization by ginsenoside Rg1 [55].

## 4. Perspective

As mentioned above, three ginsenosides were found to induce M2 polarization of macrophages or microglia, resulting in pro-resolving and anti-inflammatory effects. Based on the available literature, a possibility that more ginsenosides may induce M2 polarization was found. The assumption was based on the following indirect evidence: (1) IL-10 production or glucocorticoid receptor activation could induce M2 polarization, and (2) the anti-oxidative Nrf2–HO-1 pathway could induce M2 polarization.

Because exposure to IL-4, IL-10, or glucocorticoids could induce a type of M2-polarized macrophage [51,52], the previously reported induction of anti-inflammatory cytokine IL-10 by ginsenosides Rg6, Rb1, Rc, Rd, Re, Rf, Rg1, Rh1, Rh2, and Rp1, as well as compound K [14,23,24,41,61,62,63,64,65,66,67], or activation of glucocorticoid receptors by compound K, ginsenosides Rg1, and Re [36,37,38,39] may imply that those ginsenosides could partly induce the resolution of inflammation through M2 polarization.

Ginsenoside Ro was found not only to inhibit ROS production but also to induce HO-1 expression in RAW264.7 macrophages, with the induction of HO-1 being correlated with decreased pro-inflammatory molecules such as iNOS and COX-2 induced by LPS [33]. Because HO-1 induction could drive the phenotypic shift to M2 macrophages [68], the anti-inflammatory effect of ginsenoside Ro may also be mediated through M2 macrophage polarization.

Nrf2 is a key transcription factor to control the basal and inducible expression of more than 200 genes including antioxidants [69,70]. Nrf2 activation suppressed a set of pro-inflammatory cytokines including iNOS, MCP-1, and MIP-1β while minimally regulating NF-κB activity and the expression of its downstream cytokines, such as IL-6, IL-1β, and TNF-α in macrophages [71]. Recently, Nrf2 activation was linked to M2 polarization in macrophages [72,73]. Panaxynol, one of the major polyacetylenes, was found to be a potent Nrf2 activator, and it activated Nrf2 post-transcriptionally by inhibiting Keap-1-mediated degradation [74]. Therefore, panaxynol suppression of cytokine expression via the activation of Nrf2 may imply that its activation of Nrf2 may induce M2 polarization to exert anti-inflammation [74]. Because HO-1 is one of the target genes of Nrf2, and both Nrf2 and HO-1 could drive M2 polarization, the Nrf2–HO-1 pathway might be considered as a regulatory set for M2 polarization. Furthermore, ginsenoside Rd protected the heart against ischemia/reperfusion injury via enhanced expression of Nrf2 and HO-1 [75]. Additionally, ginsenoside Rd was found to induce CD4^+^ Foxp3^+^ CD25^+^ regulatory T-cell (Treg) differentiation by upregulating Foxp3 expression, and it increased the generation of IL-10, TGF-β1, and IL-35, suggesting that ginsenoside Rd may have the potential to modulate M2 polarization [13]. Ginsenoside Re enhanced the activation of Nrf2 in Aβ-induced SH-SY5Y cells [76]. The activation of the Nrf2–HO-1 pathway for M2 macrophage polarization is also supported by the findings that a red ginseng-derived saponin fraction suppressed inflammatory responses via the Nrf2–HO-1 pathway in an adipocyte–macrophage co-culture system [42], and that saponins from *Panax notoginseng* acted as an extrinsic regulator that activates the Nrf2 antioxidant defense system and inhibits NF-κB inflammatory signaling to attenuate LPS-induced monocyte adhesion on cerebral endothelial cells [77].

As mentioned in Section 1, ginsenosides and ginseng were extensively studied for anti-inflammatory effects in the last decade [1]. Researchers mainly focused on M1-polarized macrophages and microglia to elucidate the negative regulation of pro-inflammatory cytokine expressions (TNF-α, IL-1β, and IL-6) and enzyme expressions (iNOS and COX-2), such as LPS-stimulated RAW264.7 macrophages or BV2 microglia. The resolution of inflammation derived by M2-polarized macrophages is now emerging, with a contribution to the anti-inflammatory mechanism. It will be noteworthy to determine the merged or fused mechanisms of the ginseng anti-inflammatory system in the future.

## Abbreviations

AMPK 5’—adenosine monophosphate-activated protein kinase, ATF-2—activating transcription factor-2, COX-2—cyclooxygenase 2, FoxO1—forkhead box O1, GR—glucocorticoid receptor, HO-1—heme oxygenase-1, IFN—interferon, IL-1β—interleukin-1β, iNOS—inducible nitric oxide synthase, LPS—lipopolysaccharide, MCP-1—monocyte chemoattractant protein-1, NF-κB—nuclear factor κ-B, Nrf2—nuclear factor erythroid 2-related factor 2, PPARγ—peroxisome proliferator-activating receptor γ, ROS—reactive oxygen species, TGFβ—transforming growth factor β, TLR4—Toll-like receptor 4, TNF-α—tumor necrosis factor-α.
